# Down-regulation of *POLYGALACTURONASE1* alters firmness, tensile strength and water loss in apple (*Malus* x *domestica*) fruit

**DOI:** 10.1186/1471-2229-12-129

**Published:** 2012-08-02

**Authors:** Ross G Atkinson, Paul W Sutherland, Sarah L Johnston, Kularajathevan Gunaseelan, Ian C Hallett, Deepali Mitra, David A Brummell, Roswitha Schröder, Jason W Johnston, Robert J Schaffer

**Affiliations:** 1 The New Zealand Institute for Plant & Food Research Limited (PFR), Mount Albert Research Centre, Private Bag 92169, Auckland 1142, New Zealand; 2 PFR, Food Industry Science Centre, Private Bag 11600, Palmerston North, 4442, New Zealand; 3 The University of Auckland, Private Bag 92019, Auckland 1142, New Zealand

**Keywords:** Apple (*Malus* x *domestica*), Fruit softening, Intercellular adhesion, Polygalacturonase, Texture, Pectin

## Abstract

**Background:**

While there is now a significant body of research correlating apple (*Malus* x *domestica*) fruit softening with the cell wall hydrolase *ENDO*-POLYGALACTURONASE1 (PG1), there is currently little knowledge of its physiological effects *in planta*. This study examined the effect of down regulation of PG1 expression in ‘Royal Gala’ apples, a cultivar that typically has high levels of PG1, and softens during fruit ripening.

**Results:**

*PG1*-suppressed ‘Royal Gala’ apples harvested from multiple seasons were firmer than controls after ripening, and intercellular adhesion was higher. Cell wall analyses indicated changes in yield and composition of pectin, and a higher molecular weight distribution of CDTA-soluble pectin. Structural analyses revealed more ruptured cells and free juice in pulled apart sections, suggesting improved integrity of intercellular connections and consequent cell rupture due to failure of the primary cell walls under stress. *PG1*-suppressed lines also had reduced expansion of cells in the hypodermis of ripe apples, resulting in more densely packed cells in this layer. This change in morphology appears to be linked with reduced transpirational water loss in the fruit.

**Conclusions:**

These findings confirm PG1’s role in apple fruit softening and suggests that this is achieved in part by reducing cellular adhesion. This is consistent with previous studies carried out in strawberry but not with those performed in tomato. In apple PG1 also appears to influence other fruit texture characters such as juiciness and water loss.

## Background

Fruit softening is a complex developmental programme that involves the disassembly of various pectin and hemicellulose components of the primary cell wall, as well as alterations to cell turgor and fruit water status [1-4]. Pectin components of the cell wall are diverse, consisting of domains of contiguous 1,4-α-linked GalA residues known as homogalacturonan, and two different domains of rhamnogalacturonan [5]. Homogalacturonan-rich pectin is commonly found in the middle lamella region of the cell wall where two adjacent cells abut, and is believed to play a major role in intercellular adhesion [6,7]. During fruit ripening, homogalacturonan can be depolymerised by *endo-*polygalacturonases (PGs), cell wall-localised enzymes that cleave stretches of unesterified GalA residues and weaken the middle lamella [8]. In ripe fruit, calcium cross-links between stretches of de-methylesterified GalA residues of homogalacturonan provide the main bonding between adjacent cells [9,10].

In many species, softening during early ripening does not appear to involve pectin depolymerisation, but a correlation between PG gene expression and softening rate, together with QTL analysis, has consistently implied some involvement of PG in the softening and textural change of fleshy fruit, principally during late ripening [4,11-21]. Despite extensive study, the specific contribution of *endo*-PGs to fruit softening remains an open question - particularly since studies where PG mRNA abundance and activity were directly manipulated in transgenic tomato and strawberry fruit have produced quite contrasting results.

In tomato (*Solanum lycopersicum*), down-regulation of the ripening-specific *SlPG* gene only slightly reduced the softening of the fruit [22,23]; and over-expression of *SlPG* in the ripening-inhibited *rin* background partially restored PG activity but did not restore fruit softening [24]. Cell wall analysis subsequently showed that pectin depolymerisation during ripening was not prevented and was only slightly reduced in *SlPG*-suppressed fruit [25], presumably due to the incomplete gene silencing that left ~1% of PG activity remaining [22]. Nevertheless, these experiments were interpreted as indicating that PG-mediated pectin depolymerisation was neither necessary nor sufficient for tomato fruit softening. However, in strawberry (*Fragaria* X *ananassa*), down-regulation of the ripening-related *FaPG* gene led to significantly firmer fruit [26], suggesting that PG plays a central role in strawberry fruit softening.

Ripening in both tomato and strawberry is associated with a rapid loss of firmness producing a soft fruit. In contrast, fruit of other species such as water melon (*Citrullus lanatus*), Asian pear (*Pyrus pyrifolia*) and apple (*Malus domestica*) don’t soften to the same extent and are still crisp when ripe [27]. The role of PGs in determining crisp fruit texture has not been directly assessed. Apple possesses a PG gene (*PG1*) that increases in expression during fruit ripening and after exposure of apple fruit to ethylene and the cold temperature [28-31]. Over-expression of *PG1* in apple trees led to increased intercellular separation in leaves [32], demonstrating a role for PG in the loss of intercellular adhesion. This was also suggested by Ben-Arie *et al.*[33], who showed that application of tomato PG to apple fruit discs caused similar ultrastructural changes to those occurring in over-ripe fruit, including dissolution of the pectin-rich middle lamella. Two genetic studies have also pointed to PG as being an important determinant of apple fruit softening: a major fruit firmness-related QTL has been mapped to the *PG1* locus on chromosome 10 [15,34] and reduced levels of *PG1* expression have been correlated with firmer fruit [35,36].

In this paper we investigate the role of PG1 in determining apple fruit texture by generating antisense apple lines with the fruit-specific *PG1* gene suppressed. The fruit were phenotyped for a range of chemical, ultrastructural and biophysical features to assess changes in the cell wall, cell packing and the mode of tissue failure. The present study provides new evidence for the role of PG1 in apple fruit, and presents new opportunities for understanding physiological processes not previously linked with PG.

## Results

### Suppression of *PG1* leads to firmer apple fruit and reduced pectin depolymerisation

To directly assess the role of PG1 in apple fruit ripening, a binary vector containing a construct with the cauliflower mosaic virus 35S promoter controlling the transcription of a *PG1* transgene in the antisense orientation was assembled. Ten independent *PG1* antisense (PG1as) transgenic apple lines were generated in the cultivar ‘Royal Gala’ using *Agrobacterium*-mediated transformation [37]. These plants were grown alongside untransformed ‘Royal Gala’ control lines in a containment greenhouse.

**Figure 1:**
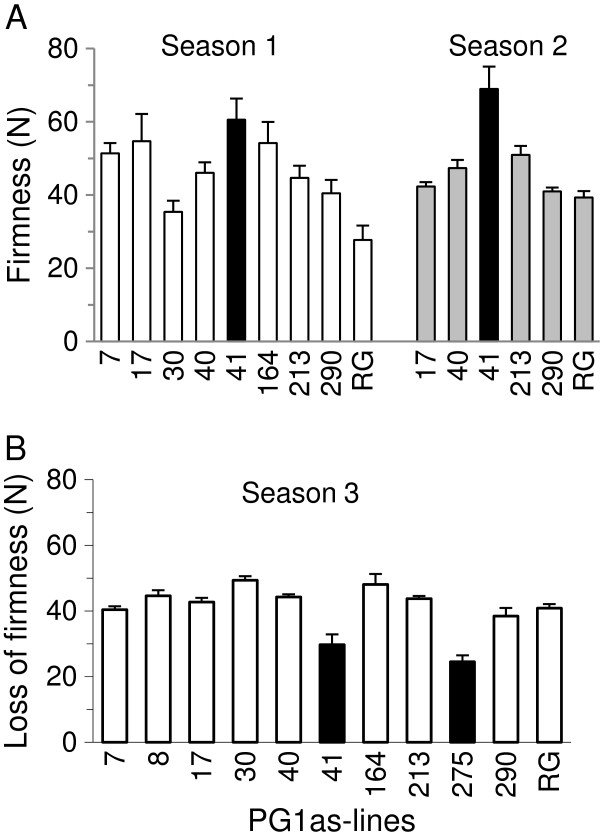
**Cortical firmness of transgenic *PG1*-suppressed and ‘Royal Gala’ control fruit harvested from three seasons.A** Cortical firmness of wild-type ‘Royal Gala’ (RG) apple fruit and transgenic *PG1*-suppressed (PG1as) lines in fruiting seasons 1 and 2. Fruit firmness was measured after ripening for 32 weeks at 5°C (n = 4–10 fruit ± Standard Error). **B** Mean loss of cortical firmness of transgenic *PG1*-suppressed and ‘Royal Gala’ fruit in season 3 after ripening for 16 weeks at 5°C. (n = 6–10 fruit ± Standard Error). Lines PG1as-41 and PG1as-275 highlighted in black showed the least change in firmness.

In the first two seasons of fruiting, only some of the PG1as lines produced small numbers of fruit (n = 4–10). Fruit were harvested when mature and assessed when ripe using a destructive puncture (penetrometric) test in the fruit cortex. One transgenic line, PG1as-41, was identified as having fruit that were consistently firmer when compared with those obtained from untransformed ‘Royal Gala’ controls and the other transgenic lines (Figure 1A).

In season 3, all ten independent PG1as lines produced fruit. Relative to controls, no differences in final fruit size or shape were observed (data not shown). Two transgenic lines (PG1as-41 and PG1as-275) were identified that were firmer after ripening than ‘Royal Gala’ controls (Figure 1B). In these two lines, softening was only ~20-30 N compared with ~40-50 N in the control and the other PG1as lines.

**Figure 2:**
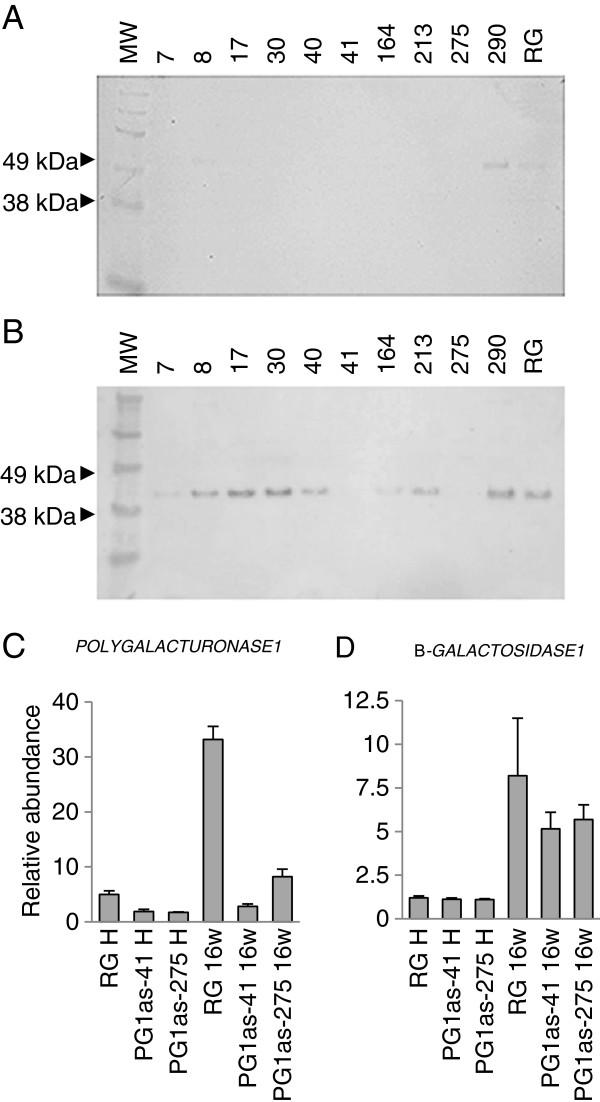
**Molecular characterisation of transgenic PG1as apple lines.** Western analysis of protein extracts from fruit of transgenic PG1as lines and ‘Royal Gala’ (RG) control after SDS-PAGE (8 μg protein per lane) at harvest **(A)**, and after ripening for 16 weeks at 5°C **(B)**, using a polyclonal antibody raised to recombinant apple PG1. mRNA abundance of *PG1***(C)***,* and a ripening-related apple β-*GALACTOSIDASE* gene **(D)**[38], at harvest (H) and after ripening for 16 weeks at 5°C (16 w). mRNA abundance of the PG1as-41 sample at harvest was set as 1 (n = 4 ± Standard Error).

For each of the transgenic lines, proteins were extracted from mature fruit at harvest and from fruit following ripening. The extracts were then screened for the presence of immunodetectable PG1 protein using protein gel blot analysis with a polyclonal PG1 antibody. At harvest, low levels of PG1 protein were detected in control fruit and one PG1as line, PG1as-290 (Figure 2A). After ripening, PG1 protein was observed at higher abundance than at harvest in the control and PG1as-290 lines. PG1 protein was also detected in seven other PG1as lines, but was undetectable in fruit of lines PG1as-41 and PG1as-275 (Figure 2B). These two independent transgenic lines with low PG1 protein abundance were also the lines with the firmest fruit after ripening (Figure 1B) and were therefore chosen for further detailed study.

In untransformed ‘Royal Gala’ controls, *PG1* mRNA abundance was strongly induced during ripening (Figure 2C). In the transgenic lines, the suppression of PG1 protein accumulation was reflected in the abundance of the *PG1* mRNA, with PG1as*-*275 and PG1as-41 fruit showing a 75% and 92% reduction in *PG1* mRNA accumulation during ripening, respectively, compared with the control (Figure 2C). As an internal ripening control, the expression of a ripening-related apple β*-GALACTOSIDASE* gene [38] was shown to be induced to a similar level in PG1as-41, PG1as*-*275 and control fruit (Figure 2D). This result suggested that other aspects of cell wall metabolism related to ripening were proceeding as normal.

**Figure 3:**
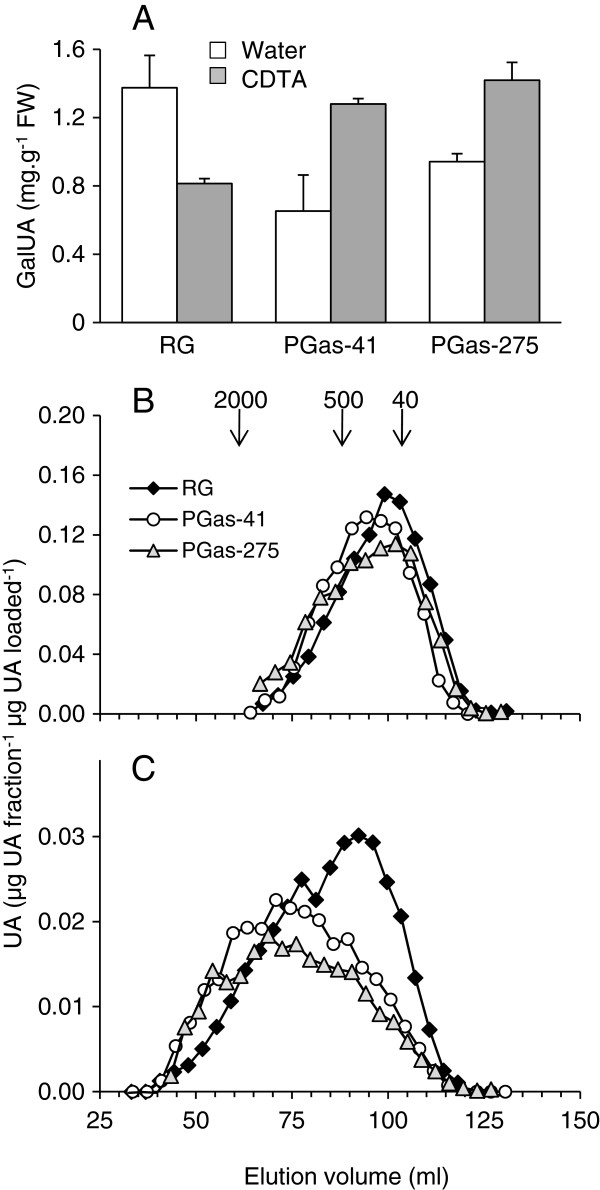
**Polyuronide content and size distribution of water-soluble and CDTA-soluble polyuronides from the cell walls of transgenic PG1as and ‘Royal Gala’ (RG) control apple fruit after ripening for 16 weeks at 5°C.A)** Polyuronide content, expressed as mg of uronic acid per g fresh weight of tissue, of water-soluble extracts (white bars) and CDTA-soluble extracts (grey bars) (n = 3 ± Standard Deviation). **B)** Size distribution of water-soluble polyuronides, and **(C)** CDTA-soluble polyuronides after elution on Sepharose CL-2B (profiles are typical examples from two extractions). Arrows indicate elution of dextran molecular weight markers (kDa).

*In vitro*, PGs hydrolyse the homogalacturonan backbone of pectin polymers with a low degree of methylesterification, and hence may play a role in pectin solubilisation and depolymerisation during fruit ripening. To determine whether there was a difference in the pectin components of the cell walls of the two *PG1*-suppressed lines compared with ‘Royal Gala’ controls, cell walls were prepared from fruit after ripening then extracted to solubilise pectins. Sequential extractions solubilised water-soluble pectin, which is essentially unattached to the cell wall, followed by CDTA-soluble pectin, which is held in the wall by calcium bonds and is attributed to pectin of the middle lamellae. Relative to controls, suppression of the *PG1* gene substantially reduced the amount of water-soluble pectin and increased the amount of CDTA-soluble pectin (Figure 3A). The polyuronide content in the water-soluble extract from the PG1as-41 line was less than half that of ‘Royal Gala’ control fruit, with PG1as-275 being intermediate, but also substantially reduced relative to the control. A corresponding increase in the amount of CDTA-soluble polyuronides was found in the two *PG1*-suppressed lines, which possessed a polyuronide content at least 50% greater than the control. These data indicate that in the *PG1*-suppressed lines pectin is less soluble than in controls, and a greater proportion is retained in the wall by calcium bonds.

Analysis of the size distribution of water-soluble polyuronides showed that the relative average molecular mass was similar between PG1as lines and ‘Royal Gala’ control, with the polyuronides of the two transgenic lines on average being only slightly larger than the control (Figure 3B). The CDTA-soluble polyuronides from both PG1as-41 and PG1as-275 were considerably larger than in the ‘Royal Gala’ control, seen as a substantial shift in the peak average molecular weight to higher molecular mass (Figure 3C).

### Suppression of PG1 reduces water loss from ripe fruit and changes the structure of hypodermal cell layers

**Figure 4:**
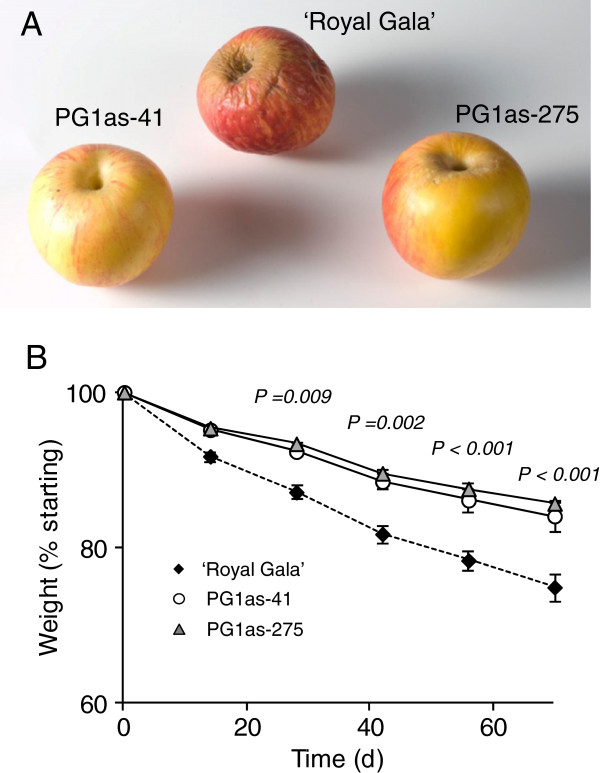
**Transpirational water loss in transgenic PG1as and ‘Royal Gala’ control fruit.** Fruit were initially ripened at 5°C for 16 weeks then transferred to 20°C for 9 weeks. **A)** Representative photograph of PG1as-41, PG1as-275 and ‘Royal Gala’ (RG) control fruit after being held for 9 weeks at 20°C. Note how the PG1as-41 and PG1as-275 fruit were substantially less wrinkled than the control. **B)** Weight loss (representing mainly water loss) was measured every 1–2 weeks for the 9 weeks fruit were held at 20°C. Weight loss is presented as a percentage of starting weight after transfer to 20°C (n = 4–6 ± Standard Error and T-test statistics between apples containing the transgene and untransformed controls are shown).

The rate of water loss in ‘Royal Gala’ control, PG1as-41 and PG1as-275 fruit was assessed at 20°C for 9 weeks following low temperature ripening. After 9 weeks, the PG1as-41 and PG1as-275 fruit showed less shrivelling compared with control fruit (Figure 4A). Whereas control fruit exhibited a relatively large mean decrease in fresh weight of 0.36% weight loss per day, the two independent *PG1*-suppressed lines showed only 0.21% (PG1as-41) and 0.23% (PG1as-275) weight loss per day (Figure 4B). This unexpected observation indicated that silencing the *PG1* gene reduced water loss from the fruit.

**Figure 5:**
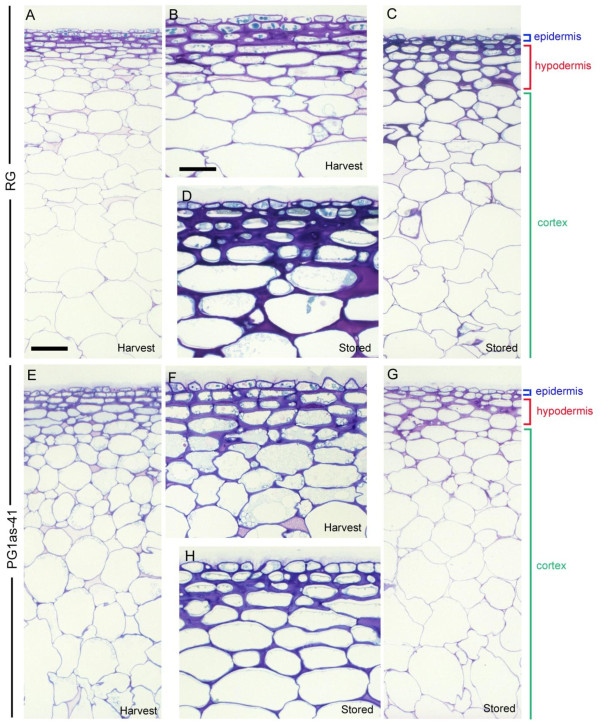
**Structural changes occurring in the exocarp and cortex of transgenic PG1as and ‘Royal Gala’ control fruit.** Toluidine blue-stained sections from: ‘Royal Gala’ (RG) at harvest **(A, B)** and following ripening for 16 weeks at 5°C - stored **(C, D)**; PG1as-41 at harvest **(E, F)** and following ripening for 16 weeks at 5°C - stored **(G, H)**. Coloured lines show the approximate extent of the epidermis/cuticle (blue), hypodermal cell layers (red) and cortex (green). Note the thickening of the hypodermal layers observed in the control (C, D) does not occur in the suppressed PG1as-41 line (G, H). Scale bars = 100 μm (A, C, E, G) and 50 μm (B, D, F, H).

To assess whether this difference in water loss was due to changes in cellular structure of the exocarp (i.e. the skin including cuticle, epidermis and hypodermis), light microscopy was conducted on fruit at harvest and following ripening. In the ‘Royal Gala’ control (Figure 5A-D), toluidine blue staining of sections of the exocarp and outer cortex showed a hypodermal layer of small cells with thickened cell walls 4–5 cells deep that extended to a depth of ~50–70 μm into the fruit (Figure 5A, D). The walls of these cells stained densely at harvest. Following ripening, the cells of the hypodermal layers in all control samples appeared to be larger and more rounded, and the cell walls (particularly in the corners) were swollen, increasing the depth of this zone to ~100 μm (Figure 5C, D). In PG1as-41 fruit at harvest, the hypodermal layer was similar to the ‘Royal Gala’ control (Figure 5E, F). However, following ripening the hypodermal layer in all samples of PG1as-41 fruit did not appear to undergo the same level of cell swelling, maintaining a more ‘at-harvest’ appearance than the ripened ‘Royal Gala’ control (Figure 5G, H).

### Suppression of *PG1* in the fruit cortex changes intercellular adhesion and alters fracture properties

**Figure 6:**
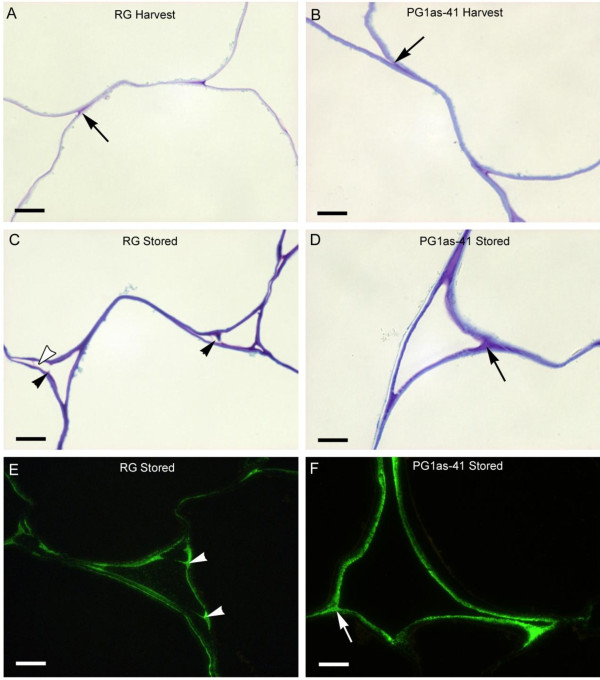
**Intercellular adhesion in the cortex tissue of ‘Royal Gala’ (RG) control (A, C, E) and transgenic PG1as-41 (B, D, F) fruit.** Toluidine blue-stained sections of cell wall boundaries at harvest **(A, B)**; and following ripening for 16 weeks at 5°C - stored **(C, D)**. Immunolocalisation of a pectin epitope with a low degree of esterification in the cell walls of apple fruit after ripening for 16 weeks at 5°C visualised using the antibody JIM5 **(E, F)**. Arrows indicate materials filling the space where two cells join (A, B, D, F). In (C) and (E), solid arrowheads indicate remnants of cell wall material protruding into the intercellular space. In (C) the open arrowhead indicates a region where material has been lost between adjacent cells. Bar = 10 μm.

Underneath the hypodermal layers, apple cortical cells are larger and rounder than near the epidermis and there are more visible intercellular spaces (Figure 5A). At harvest, the cell walls in this region stained similarly in both the ‘Royal Gala’ control and PG1as-41 fruit. Cell walls typically stained evenly, except at the junctions between three cells, or between two cells and an intercellular space where staining was more intense. There was no sign of cell-to-cell separation in either controls or in *PG1*-suppressed fruit (Figure 6A, B). Following ripening, cells in the ‘Royal Gala’ control showed separation at the corners of attachment between three cells and along the middle lamella (Figure 6C). Once the corners had separated, two or three remnants of wall material protruded into the intercellular space. In the PG1as-41 fruit, neither separation of the corners nor protruding cell wall remnants were observed (Figure 6D).

Immunolabelling of ripened ‘Royal Gala’ cortex tissue with the JIM5 antibody (recognising homogalacturonan with a low degree of methylesterification) showed labelling along the cell wall, and strong labelling of the cell wall protrusions (Figure 6E). In ripened tissue from the PG1as-41 line, labelling was evenly spread over the cell wall and no cell wall protrusions were observed (Figure 6F). These changes in cell separation and immunolabelling suggested that there might be a change in intercellular adhesion properties that could be measured using a tensile test (force needed to pull tissue apart) rather than a puncture test (mixture of compression and shear forces).

**Figure 7:**
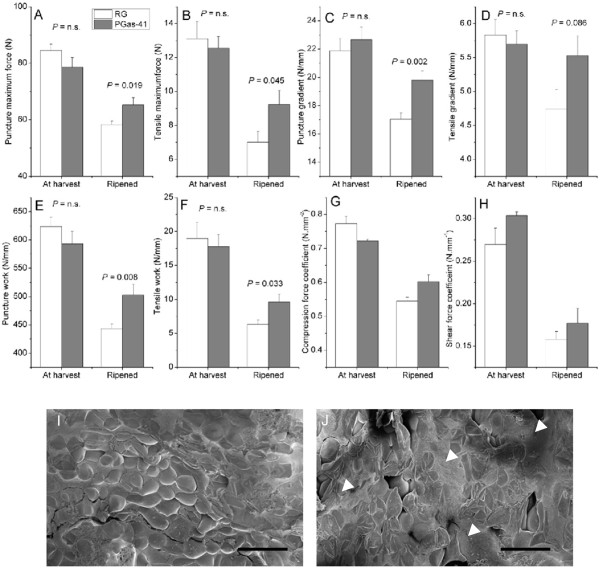
**Differences in puncture maximum force (A), tensile maximum force (B), puncture gradient calculated from stress–strain curve (C), tensile gradient calculated from stress–strain curve (D), puncture work calculated from area of stress–strain curve (E), tensile work calculated from area of stress–strain curve (F), compression force coefficient (G), and shear force coefficient (H), between transgenic PG1as-41 and ‘Royal Gala’ (RG) control fruit at harvest and following ripening at 0.5°C for 10 weeks (n = 15 ± Standard Deviation).** Statistics for A-F were performed using a T-test. The coefficients and standard errors in (G) and (H) were determined using non-linear regression. Scanning electron microscope views are of a ‘Royal Gala’ section (I) and a PG1as-41 section (J) taken after tensile tests from fruit following ripening. White arrow heads show the presence of ruptured cells. Scale bar represents 500 μm.

To test this hypothesis, in season 4, ‘Royal Gala’ control and PG1as-41 fruit were re-assessed using puncture and tensile tests at harvest and following a shorter ripening treatment (10 weeks, 0.5°C). At harvest, there were no significant differences in maximum puncture firmness (Figure 7A) or maximum tensile force between the ‘Royal Gala’ control and PG1as-41 apples (Figure 7B). In contrast, in ripened fruit, maximum puncture firmness and maximum tensile force were both significantly higher in PG1as-41 apples compared to the ‘Royal Gala’ control (Figure 7A, B). Force deformation analysis showed that the ripened PGas-41 apples had a higher Young’s modulus (gradient of stress–strain curve) and higher work (area under stress–strain curve) for both puncture and tensile tests when compared to ‘Royal Gala’ control apples (Figure 7C-F). However these two differences in mechanical properties correlated with fruit firmness, with apples of similar firmness showing the same mechanical properties regardless of the presence of the transgene. Different probe sizes were also used to assess the relative contributions of shear (perimeter) and compression (area) forces during the puncture test. This approach showed that the compression forces were dominant and closely reflected the maximum firmness values (Figure 7G). In contrast, while a more minor component, the shear forces were higher for the PG1as-41 apples both at harvest and after ripening (Figure 7H). This was the only mechanical feature which was higher in the PG1as-41 apples at harvest.

Sections of ripened fruit after the tensile strength tests were snap frozen in liquid nitrogen and examined on a cold stage scanning electron microscope (SEM). The fracture surfaces of the pulled-apart sections of ‘Royal Gala’ showed areas of undamaged, un-ruptured rounded cells and low juice (Figure 7I, Additional file 1: Table S1), whereas sections from PG1as-41 showed a relatively large number of damaged (deflated) cells and sheet-like layers of juice obscuring cortex cells, indicating more cellular fractures and ruptures, and more free fluid in the cortex tissue (Figure 7J, Additional file 1: Table S1).

## Discussion

Historically, apple has been reported to lack detectable depolymerisation of pectin during ripening [39]. Here we show that suppression of apple *PG1* results in a clear increase in the average molecular mass of pectin, particularly in a CDTA-soluble fraction. Since *PG1*-suppressed fruit were significantly firmer than control fruit, it appears that the molecular weight distribution of pectin influences the firmness of apple fruit, presumably through increased integrity of the middle lamella. This result is consistent with data concerning the down-regulation of *FaPG* and pectate lyase in transgenic strawberry lines [26,40], but not with those regarding down-regulation of *SlPG* in transgenic tomato lines. The reason for this difference may relate partly to the degree of PG down-regulation achieved in tomato, and partly to the system studied. There are large differences in the extent to which depolymerisation naturally occurs in these fruit; pectin depolymerisation in both apple and strawberry is slight, whilst in tomato it is more extensive [2]. Tomato possesses unusually high levels of PG activity, and the ~1% of activity remaining in *SlPG*-suppressed fruit was apparently sufficient to cause almost wild-type levels of pectin depolymerisation [25]. Based on the transgenic work in strawberry and that presented here, it thus seems likely that PG-mediated pectin depolymerisation provides a component of the softening seen in many ripening fruit.

Sensory studies have shown that upon biting and chewing, the primary walls of apple fruit rupture and the cellular juices are released, resulting in juiciness [41]. The link between sensory juiciness and free fluid in pulled-apart sections in apple has been correlated with differences in pectin composition of which the most notable is a reduced galacturonic acid content in the water-soluble pectin fraction [42]. In this study, assessments of tensile properties and shear forces during puncture testing in conjunction with scanning electron microscopy showed that ripening in apple is normally accompanied by decreasing strength of the middle lamellae, resulting in reduced intercellular adhesion and cell separation under stress. In *PG1*-suppressed tissue, middle lamella strength was retained and the point of failure under stress became the primary cell walls, resulting in cellular rupture and release of free fluid. These higher levels of free fluid and increased tensile strength and shear forces indicate potential for a fruit with a juicier, crisper texture.

An unexpected consequence of *PG1* suppression that has emerged from this work is a change in hypodermal anatomy that may influence fruit firmness by regulating water status. Declining cell turgor is a normal part of fruit softening [1,43-45] and is brought about by a combination of the movement of osmotic solutes and water to the apoplast [46-48] and by transpirational water loss from the fruit [3]. In tomato, the lack of softening in the *DFD* mutant was attributed to very low water loss and increased cellular turgor [3]. The data of Figures 4 and 5 show not only that an increase in transpirational water loss from the fruit is a part of ripening in apple, but that this is at least in part controlled by developmental changes in the hypodermal layers brought about by the action of PG. In control fruit, a swelling of the hypodermal cell layers occurred during ripening, and was correlated with increasing whole-fruit water loss. In the *PG1*-suppressed lines, cells in the hypodermal layers of the fruit below the cuticle remained densely packed, which was correlated with slower water loss from the fruit and reduced shrivelling. The cuticle and surface cell layers of fruit are known to change both in wax composition and in thickness and structure during ripening [49,50]. This can include a reduction in the amount of cell wall polysaccharide that is associated with the cuticle [51]. In apple, although the surface conductance declines from an initial high level in developing fruit, the rate of water loss per fruit declines only slightly by the time fruit are ripe [52]. The data presented here suggest that the action of PG may indirectly promote fruit water loss during ripening, in part by reducing intercellular adhesion and allowing a swelling of the hypodermal cell layers (Figure 4C).

A relationship between pectin fine structure and tissue anatomy has been shown both in fruit and in other organs. In potato tubers, fragmentation of rhamnogalacturonan I by a fungal rhamnogalaturononan lyase induced radial swelling of the periderm cells and the development of cortical intercellular air spaces [53]. In tomato fruit, down-regulation of the ethylene-regulated auxin response factor DR12 reduced pectin solubilisation and altered the distribution of methyl ester side groups, which produced a higher proportion of small cells in the outer pericarp and a thinner cuticle, and fruit firmness was increased [54]. Interestingly, over-expression of a fungal PG in tobacco altered cellular arrangement and enhanced fresh weight loss from detached leaves [55], suggesting that PG may play a wider role in altering intercellular adhesion that can affect cell shape and packing, which in some tissue results in increased water loss.

## Conclusion

By comparing transgenic apple lines that differ only in the expression of a single gene, we show for the first time the effects of PG1 action on apple cell wall metabolism during ripening. Our data confirm PG1’s role in apple fruit softening and textural change and suggest that this is achieved in part by cell wall disassembly and loss of intercellular adhesion. The unexpected effects of *PG1* suppression on fruit anatomy and transpirational water loss show that *PG1’s* function in fruit ripening is more complex than previously reported.

## Methods

### Generation of transgenic plants and plant growth

A cDNA derived from the fruit-specific apple polygalacturonase gene *MdPG1*[32] was ligated in the antisense orientation into pART7 to produce a *35S:PG1-as:nos* cassette, and then the cassette was ligated into the *Not*1 site in the pART27 binary vector [56]. pART27-PG1as was introduced into *Agrobacterium tumefaciens* strain LBA4404 by electroporation, and used to generate 10 independent transgenic apple (*Malus domestica* ‘Royal Gala’) plants [37]. Transgenic plants were grown in a containment greenhouse alongside untransformed ‘Royal Gala’ controls. Plants were grown under natural daylight and temperature conditions in 50 L planter pots. Plants received 8 weeks winter chilling at 7°C each year to promote and synchronise flowering. Flowers were hand pollinated each spring using compatible ‘Granny Smith’ pollen. Wild-type and *PG1*as knockdown trees were managed in the same way by thinning fruit to one/two fruit per cluster, with trees having a similar TCA (trunk cross-sectional area). Trees typically carried between 10–30 fruit per plant.

### Force deformation analysis during ripening

Fruit were harvested when mature based on background skin colour and starch pattern index [57]. All misshapen, marked or damaged fruit were discarded. Pedicels were removed from all fruit which were then placed in single layer plastic trays under polyethylene plastic liners in commercial cardboard cartons. Fruit were subjected to low temperature ripening in a constant temperature room at 5°C for 16 weeks and a relative humidity of 80-85%, unless stated otherwise. A cold treatment was used, since expression of the apple *PG1* gene increases strongly in wild-type fruit after exposure to cold [28] thereby accentuating the physiological effects of altering PG levels in control vs. transgenic fruit. Furthermore, the exposure of apples to cold can be important for ensuring the ethylene climacteric and ripening proceeds fully [58]. The requirement for cold exposure in the present study is also accentuated by the apples being grown in a greenhouse and having less cold night conditioning before harvest than would normally occur in the field.

Mechanical properties during tissue failure were assessed using puncture [57] and tensile tests ( [59]. A puncture test was performed by driving cylindrical probes (2, 5, 8 and 11 mm diameter) into the flesh at a constant speed of 4 mm·s^-1^ to a depth of 9 mm using a TA.XT*Plus* Texture Analyser (Stable Microsystems, United Kingdom). All assessments were performed following the removal of the skin (~1-2 mm deep - including cuticle, epidermis and hypodermis) from two opposing locations on the fruit equator. Multiple probe sizes were used to minimise complications associated with performing puncture tests on small fruit and to allow estimates of puncture and shear coefficients [60]. Tensile properties were measured using an excised block of fruit tissue (10 mm × 4 mm), with notches cut at each side through the middle to provide a weakened zone and allow the tissue to be pulled apart with metal claws attached to the TA.XT*Plus* Texture Analyser. The claws moved apart at a constant rate of 10 mm·min^-1^ until tissue failure. For both tensile and puncture tests, full force deformation analysis was done to determine Young’s modulus (slope before failure), maximum force, distance at maximum force, and work (area under curve) [61].

### Antibody generation, protein extraction and western analysis

The mature ORF of apple *PG1* was amplified using primers RA136 5’- ACGGGATCCG CTCCGGCCAA AACCATTAGC-3’ and RA137 5’- ATAGTTTAGC GGCCGCTTAA CATCTAGGGG AGACAAC-3’. The insert was excised with *Bam*HI and *Not*I (underlined in the primers) and ligated into corresponding sites of the pET-30a(+) vector (Novagen, Madison, WI, USA) to produce plasmid pETPG1. pETPG1 was transformed into *Escherichia coli* BL21 cells containing the pLysS plasmid and recombinant His-tagged protein was purified by Ni-affinity chromatography under denaturing conditions [62]. Fractions of the eluate were separated on 10% (w/v) polyacrylamide SDS-Tris-Tricine gels and recombinant protein determined by the presence of a major band at ~37 kDa. The main band was cut out and used to raise polyclonal antiserum in rabbits (AgResearch, New Zealand).

To determine the presence of PG protein in apple fruit, frozen powdered tissue (500 mg) was mixed with 50 mg PVPP and extracted with 2 volumes of ice cold low salt buffer (0.2 M Na acetate, 10 mM DTT, 50 mM NaCl, pH 4.7) and allowed to thaw on ice for 10 min before centrifugation for 10 min, 4°C 16000 × g. The extraction was repeated again using 1 volume of ice cold low salt buffer. Two volumes of high salt buffer (0.3 M MES, 10 mM DTT, 0.6 M NaCl, pH 6.0) was mixed with the pellet and incubated on ice for 15 min before centrifugation was repeated. The supernatant was retained and the pellet extracted again with 1 volume of high salt buffer. The two high salt protein extractions were combined and protein concentrations in each sample were measured using a Protein Assay Kit (Bio-Rad Laboratories, Auckland New Zealand) using bovine serum albumin as a standard. Concentrations were verified on gels by Coomassie staining.

Proteins were separated on 12% (w/v) polyacrylamide SDS-Tris-Tricine gels using a Mini-PROTEAN3 electrophoresis system (Bio-Rad Laboratories, Auckland New Zealand), electroblotted onto polyvinyldifluoride membrane, and blocked as described by Atkinson *et al.*[32]. Proteins were immunodetected using the antiserum raised to apple PG1, which was diluted (1:1000, v/v) in TBS buffer containing 5% non-fat dried milk. Membranes were incubated with an anti-rabbit secondary antibody conjugated to alkaline phosphatase (Sigma-Aldrich), and binding to PG1 was visualised using 1-Step NBT/BCIP (Pierce).

### Real-time qPCR

Total RNA was extracted from apple tissue of the ‘Royal Gala’ control and PG1as-41 and PG1as-275 lines as described in Chang *et al.*[63]. cDNA synthesis and qPCR were performed as described in Tacken *et al.*[28]. qPCR primers used for assessment of mRNA abundance of *PG1* and the actin reference gene were as described by Tacken *et al.,*[28]. Primers for the ripening-related apple β-galactosidase gene [38] were MdBGALF 5’-GCCATCAAGT CATGCGAGTA-3’ and MdBGALR 5’-CGTGTCATAG TTTGCGAGGA-3’.

### Cell wall analyses

Cell walls were prepared and extracted as described in Melton and Smith [64]. Apple cortex was snap frozen in liquid N_2_, ground to a fine powder and buffer-saturated phenol (UltraPure^™^, Invitrogen) added. The slurry was homogenised using a Polytron, centrifuged, the supernatants recovered, and the residue washed twice in H_2_O to collect water soluble polysaccharides. Supernatants were combined, dialysed and freeze-dried to give the water soluble fraction. The pellet was extracted twice with DMSO to remove starch, and the supernatants recovered, dialysed and freeze-dried to give the DMSO-soluble extract. The pellet after DMSO extraction was dialysed and freeze-dried to give the cell wall material (CWM). An aliquot was extracted with CDTA (trans-1,2-diaminocyclohexane-N,N,N’N’-tetraacetic acid) in sodium acetate, pH 6.0, and the recovered supernatants were dialysed against ammonium acetate buffer (pH 5.2) for five days, followed by H_2_O, and freeze-dried to give the CDTA-soluble extract.

Extracts were analysed for uronic acid content after Blumenkrantz and Asboe-Hansen [65] using D-galacturonic acid (Sigma) as a standard. Water- and CDTA-soluble fractions were separated by size exclusion chromatography using a column of Sepharose CL-2B (1.6 × 90 cm; Pharmacia Biotech) eluted with 0.2 M ammonium acetate, pH 5.0, at a flow rate of ~6 ml h^-1^ and a fraction size of 20 min. The polyuronide content of the column fractions was determined as above. The column was calibrated with dextran molecular weight standards of 2000, 500 and 40 kDa (Pharmacia, Uppsala, Sweden).

### Structural analyses

Multiple apples (n ≥5) that were matched for size and ripeness were selected for structural analyses. Pieces of apple fruit tissue 5 mm^3^ were fixed in either 2% (v/v) formaldehyde with 0.1% (v/v) glutaraldehyde in 0.1 M phosphate buffer, pH 7.2, or 2% (v/v) formaldehyde with 2.5% (v/v) glutaraldehyde in 0.1 M phosphate buffer, under vacuum for 1 h, washed in buffer, dehydrated with an alcohol series, and embedded in LR White resin [66]. Embedded material was sectioned at a thickness of 1 μm and stained with 0.05% (w/v) toluidine blue in borate buffer (pH 4.4) for gross cytological examination and at a thickness of 200 nm for immunolabelling [67]. The thin sections were antibody-labelled as described in Sutherland *et al.*[68]. Light microscopy was carried out on an Olympus Vanox AHT3 compound microscope (Olympus, Tokyo) with image recording by a CoolSnap digital camera (Roper Scientific, Tucson, AZ). Multiple sections (n ≥20) from multiple tissue blocks (n ≥2) were examined to obtain representative views.

Fracture surfaces from fruit after tensile testing were observed using Cryo-Scanning Electron Microscopy [41]. Blocks of tissue 4–5 mm high were cut from the fracture face to fit into 8 mm diameter shallow copper dish holders and secured with tissue freezing medium (Triangle Biomedical Sciences, Durham, NC) for adhesion. The fracture face protruded above the surface of the holder. The assembly was rapidly frozen in liquid N_2_ and stored in containers under liquid N_2_. Prior to observation, the holder was transferred under N_2_ to a copper specimen mount of a Sputter Cryo system (model SP 2000, Emscope, Ashford, UK) and then moved in a dry argon atmosphere to the specimen chamber of the Sputter Cryo. The sample was sputter-coated with gold at a temperature less than −130°C. The sample was transferred under vacuum to a cryo-stage (temperature −150°C or lower) in a PSEM 505 scanning electron microscope (Philips, Eindhoven, The Netherlands), where it was observed using an accelerating voltage of 12 kV. Digital images were collected using an ADDAII digital interface and Scandium software (Olympus Soft Imaging Solutions, Münster, Germany).

## Abbreviations

PG1: ENDO-POLYGALACTURONASE1; CWM: Cell Wall Material; cDNA: Complementary DNA; qPCR: Quantitative Polymerase Chain Reaction.

## Competing interest

The authors declare that they have no competing interests.

## Authors’ contributions

RGA conceived of the project, DM made the transgenic plants, PWS and ICH undertook the microscopy, SLJ and RS undertook cell wall composition assessments, KG did fruit assessments and molecular and protein characterisation, RGA, RS, JWJ, and RJS designed the experiments and were involved in the data analysis, RGA, RS, DAB, ICH, JWJ and RJS wrote the paper. All authors read and approved the final manuscript.

## Supplementary Material

Additional file 1 **Table S1.** Assessment of cell integrity and juice in pulled-apart sections. Click here for file
