# Supplementary material for: Down-regulation of POLYGALACTURONASE1 alters firmness, tensile strength and water loss in apple (Malus x domestica) fruit
Source: BMC Plant Biol. 2012 Aug 2;12:129. doi: 10.1186/1471-2229-12-129 (PMC3509026; doi:10.1186/1471-2229-12-129)
Supplement: Additional file 1 — Table S1. Assessment of cell integrity and juice in pulled-apart sections. [file 1471-2229-12-129-S1.doc]

**Table S1.** Assessment of cell integrity and juice in pulled-apart sections.

|  | ‘Royal Gala’ | | | | |  | PG1as-41 | | | | |
| --- | --- | --- | --- | --- | --- | --- | --- | --- | --- | --- | --- |
| Firmness  (N) | | Pulled | Ruptured | Fractured | Juice | Firmness  (N) | | Pulled | Ruptured | Fractured | Juice |
| 66-68 | | **+½** | **++** | **+** | **++½** | **65-68** | | **+½** | **++** | **+½** | **+++** |
| **56-57** | | **+½** | **+½** | **+** | **+** | 56-57 | | + | **++** | **+** | **++½** |
| 53-54 | | **+½** | ++ | **½** | ++ | 52-55 | | **+½** | +**½** | **+½** | **++½** |

The surfaces of pulled-apart sections of ‘Royal Gala’ control and PG1as-41 fruit with similar firmness were visually assessed over multiple different SEM views (n ≥10) for integrity of cells on the surface and the amount of free juice. For each firmness range, sections were obtained from two ‘Royal Gala’ control and two PG1as-41 apples. The integrity of cells was visually assessed as ‘pulled’ – separated along the cell-to-cell boundary and still maintaining a rounded appearance; ‘ruptured’ – where cells were deflated with visible damage; or ‘fractured’ – where the fracture plane has cut across the cell. The amount of free juice was assessed by the presence of flat juice surfaces between the cells. Bold indicates typical firmness of fruit ripened for 10 weeks at 0.5°C.
